# Aspects of Equality in Mandatory Partnerships – From the Perspective of Municipal Care in Norway

**DOI:** 10.5334/ijic.2025

**Published:** 2016-05-18

**Authors:** Ralf Kirchhoff, Birgitte Ljunggren

**Affiliations:** Associate Professor, Trøndelag R&D Institute, Steinkjer, Norway; Senior Researcher, Trøndelag R&D Institute, Steinkjer, Norway

**Keywords:** The Norwegian Coordination Reform, partnership, equality, mandatory agreements

## Abstract

**Introduction:**

This paper raises questions about equality in partnerships, since imbalance in partnerships may effect collaboration outcomes in integrated care. We address aspects of equality in mandatory, public-public partnerships, from the perspective of municipal care. We have developed a questionnaire wherein the Norwegian Coordination Reform is an illustrative example. The following research question is addressed: What equality dimensions are important for municipals related to mandatory partnerships with hospitals?

**Theory/methods:**

Since we did not find any instrument to measure equality in partnerships, an explorative design was chosen. The development of the instrument was based on the theory on partnership and knowledge about the field and context. A national online survey was emitted to all 429 Norwegian municipalities in 2013. The response rate was in total 58 percent (n = 248). The data were mainly analysed using Principal component analysis.

**Results:**

It seems that the two dimensions “learning and expertise equality” and “contractual equality” collects reliable and valid data to measure aspects of equality in partnerships.

**Discussion:**

Partnerships are usually based on voluntarism. The results indicate that mandatory partnerships, within a public health care system, can be appropriate to equalize partnerships between health care providers at different care levels.

## Introduction

Several authors have pointed to the partnership “imperative” [1,2,3], and the difficulty of finding a contemporary policy document that does not have partnership as the central strategy for the delivery of care. As such, partnerships have become a popular governing tool to enhance collaboration and cooperation across boundaries in public services as part of the post New Public Management reforms [3,4,5]. This paper raises questions about equality in mandatory partnerships, since imbalances in partnerships may affect collaboration outcomes in integrated care [4,6]. The aim of this paper is to describe aspects of equality in mandatory, public-public partnerships, from the perspective of municipal care (in this paper used interchangeably with primary health care). We have developed a questionnaire wherein the Norwegian Coordination Reform [7] is an illustrative example. Inequality in relation to hospitals was perceived as one of the major barriers to achieve collaboration outcomes, before the Norwegian Coordination Reform [8]. The following research question is addressed: What equality dimensions are important for municipals related to mandatory partnerships with hospitals?

Revealing aspects of equality in partnerships at the organizational level will hopefully, in ongoing and future research be useful to interpret to what degree such factors influence outcomes. Firstly, the theoretical framework and background will be presented, with a focus on mandatory partnerships. We then describe the explorative research design and data of this study. In the method section, we describe how we used Principal component analysis [9] to explore different equality aspects in partnerships. After the presentation of the results, the discussion will focus on implications, limitations and suggestions for further research.

## Mandatory partnerships in health care

In many high-income countries, integration of health services is hindered by the fragmented supply of health services as a result of specialization, differentiation, segmentation and decentralization [10]. Given these challenges, collaboration in primary health care services has been at the forefront of the Norwegian policy agenda and the WHO [11,12]. Hence, in Norway mandatory partnerships in conjunction with other reform tools are now being used to achieve structural changes in the delivery and organization of its health care services [13].

In the organizational and integrated care literature, partnerships have been described as networks, inter-organizational relations, coalitions and strategic alliances of organizations [14,15,16]. Some of the discussions around partnership involve discussion over whether partnerships are synonymous with a particular mode of governance (i.e., network, hierarchy or market) [10,17]. In practice, however partnership has been implemented as all of these different modes and through a range of different means [15]. In its most general terms, a partnership is an agreement between two or more parties. However, many partnerships both in health and social care are formal and often require a written agreement or contract that defines the reciprocal rights and obligations of each party, the objectives of the partnership, and how the partnership will be evaluated or regulated [3,4,18,19]. For the purpose of our analysis, the OECD’s (1990) definition of partnerships is useful:

“[Partnerships are]…… systems of formalized co-operation, grounded in legally binding arrangements or informal understandings, cooperative working relationships, and mutually adopted plans among a number of institutions. They involve agreements on policy and program objectives and the sharing of responsibility, resources, risks and benefits over a specified period of time” [20, p. 19].

In line with this definition, the “litmus test” for identifying a partnership is to check whether we can identify a network of interdependent yet autonomous actors engaged in institutionalized processes of public governance based on negotiated interactions and joint decision making [16]. Theoretically, pros and cons of partnerships have been discussed in literature [1,2,3,4,21,22]. According to Dowling et.al. [4] literature has conceptualized the success of partnerships in two main ways: (1) process issues, such as how well the partners work together in addressing joint aims and the long-term sustainability of the partnership; and (2) outcome issues, including changes in service delivery, and subsequent effects on the health or well-being of service users. Yet, there has been a lack of empirical research to show that health care organizations or users benefit from partnerships [4,6].

The benefits of partnerships in policy documents tend to neglect some partnership problems and limitations: 1) that they are not always based on voluntarism, but also on reluctance. The approach to partnerships in policy documents is often normative and describe what ideal partnerships ought to be [1,2,3]. This approach denotes a high degree of volunteerism and interdependence [19,20]. Yet, there are several examples of partnerships in health care that are not strictly voluntary, but imposed through legislation by state government [23,24,25]. 2) They are not necessarily based on equal mutual dependence. This may affect equality issues in partnerships, especially for the most dependent part. The variation of dependency may be rooted in the partners’ different access to resources. Vital resources in such partnerships might be financial strength, information, equipment, capacity, time, expertise and definition power [26,27]. Resources are a basis of power for organizations [23], also in mandatory networks [27], and may have an impact on mutuality and authority in decision making or negotiations [27,28]. A focus on potential inequalities in partnerships and non- voluntarism distinguishes our perspective from an ideal-type partnership model where all partners have an equal opportunity to influence shared objectives, processes, outcomes and evaluation, despite of imbalance in power [19].

## The example: primary health care in Norway and mandatory partnerships

The example used in this article is the Norwegian Coordination Reform [7] and the subsequent negotiation of mandatory partnership. The Norwegian Coordination Reform resembles similar health care reforms introduced in other industrial countries [24,29], for example the Structural Reform in Denmark [23]. Similar to the Structural Reform, the Norwegian Coordination Reform introduced mandatory partnerships in conjunction with other reform tools to achieve structural changes in the delivery and organization of its health care services [11,13]. Some remarks about the national context are nevertheless required.

In Norway local authority, the municipality, is responsible for the provision of all primary health care services [11]. The parliament can only regulate local authorities (municipalities) by law, although financial instruments, technical guidance and various action plans are used to influence the quality of services provided [11]. Regional Health Authorities, owned by the national government, are responsible for the provision of specialized health care services [11]. In the continuation of this paper, specialized health care service refers to medical, professional and administrative services delivered by public hospitals. The range and depth of the municipalities’ responsibilities for health care have increased, as a result of the Norwegian Coordination Reform, introduced January 2012 and directed towards co-operation, prevention, self-management, treatment and rehabilitation [11]. This current reform policy in primary health care, advocates a shift towards mandatory, formalized partnerships, and more responsibilities for municipalities [7,11,30].

There was a widespread use of various more or less formalized partnerships between hospitals and municipalities in Norway [8] before the Norwegian Coordination Reform and the introduction of mandatory partnerships. Research suggests that it was challenging to achieve desirable benefits for both municipal care and hospitals [8,31]. Collaboration in these partnerships in Norway, may be hampered by factors such as differences in equality related to knowledge and administrative/economic power [27]. The hospitals were perceived as having both a professional and organizational power of definition [8,32]. The hospitals were regarded to be the strongest partner [7]. Professional and organizational solutions were often marked by the interests of the hospitals [8]. Halting collaboration was often found in situations of patient discharge or admittance, obstructing a functioning, collaborative chain [13,32]. To promote mandatory collaboration between municipal care and specialized health care, the government approved The law on health and long-term care in June 2011 [33]. The law introduced mandatory service agreements between Norwegian municipalities and hospital trusts through four Regional Health Authorities. This law specifies several areas where municipalities and hospitals are ordered to establish service agreements [33]. The law describes agreements for discharges from hospitals for patients that need further treatment in primary health care, agreements for mutual knowledge sharing, and electronic exchange of patient information.

Seen from the perspective of the municipality numerous equality aspects may result in relative success or failure in mandatory collaboration with hospitals. Possible equality aspects we focus on in this paper are perceived equality in negotiating situations, and perceived equality related to mutual learning and knowledge sharing. These equality aspects are not chosen at random, but are embedded in organizational and integrated care literature [4,10,17,28,34,35].

## Research design and data

We chose an explorative design to understand the complexity of equality in partnerships from the viewpoint of Norwegian municipalities in relation to the hospitals. We did not identify any validated instrument to measure various dimensions of equality in partnerships, hence we constructed an instrument based on theory [14,19,22] and knowledge about the field and context. To safeguard and improve validity the survey was first developed and reviewed by a multidisciplinary research group and then piloted on a sample of eight municipal health care managers. Based on feedbacks from the latter, followed by separate interviews with each pilot respondent, the questions in the survey were adjusted.

The target group was administrative health care managers with responsibility for health care in their municipality. We expected the head of the health office in the municipality to have an overall view over formalized activities within the hospital sector. Based on our understanding of the negotiation processes gained in the project, we expected them to have insights into, or having participated in, the development and implementation of mandatory service agreements.

A national online survey was emitted to all 429 Norwegian municipal in the period 26.11–18.12 in 2013 [36]. Additional 15 boroughs (city districts) in Oslo were included in the survey. We asked the municipalities to forward the survey to the person who had the highest responsibilities for municipal health care. After two reminders we received responses from 259 municipal care managers in 248 municipalities. The response rate was in total 58 percent (n = 248). The dropout analysis revealed no systematic bias between non-responders and responders [36]. Characteristics of the sample at baseline are listed in Table 1.

**Table 1:** Characteristics of sample at baseline.


Municipalities participating Response rate, %	n = 248 58 (248 out of total 429 municipalities)
Size (inhabitants in municipalities), % 0–4 999 5000–19 999 20 000 and more	49 38 13
Gender of administrative municipal health care manager, %	Male 30 (n = 175) Female 68 (n = 78) Missing 2 (n = 6)


A covering letter accompanied the online survey, explaining the purpose of the research project. The letter was based on the guidelines from the Data Protection Official for Research for all the Norwegian universities, university colleges and several hospitals and research institutes. Ethical approval of the project was obtained from the same authorities.

The study is part of a national research project called “Collaboration and integrated care pathways in the melting pot” (Samhandling og pasientforløp i støpeskjeen). The project is financed by the Norwegian Research Council. The primary objective of the project is to evaluate aspects of the Norwegian Coordination Reform, especially on mandatory service agreements and their possible impact on collaboration-outcomes between primary care and specialist health care.

## Methods

### Step 1

Principal components analysis (PCA) was used to identify possible dimensions of equality in partnerships. The objective of PCA is to extract the maximum variance of the data set with each component. PCA is a recommended solution for researchers who are primarily interested in reducing a large number of variables down to a smaller number of components [9]. 17 items from the survey were subjected to PCA using IBM SPSS version 21. Prior to performing PCA, the suitability of the data was assessed. Inspection of the correlation matrix revealed the presences of many coefficients ≥ .3. The Kaiser-Meyer-Olkin value was .79, exceeding the recommended value of .6 [9] and Bartlett’s Test of Sphericity reached significance, supporting the factorability of the correlation matrix. PCA revealed the presence of two components with eigenvalues exceeding 1, explaining 47.5 % and 19.4% of the variance respectively (Table 2).

**Table 2:** Eigenvalues, percent of variance and cumulative percent of variance.

Component	Total	% of Variance	Cumulative % of variance

1	3.799	47.486	47.486
2	1.553	19.415	66.901

An inspection of the scree-plot revealed a clear break after the second component, which was supported by additional Parallel analysis where we tested our results against a randomly generated data matrix of the same size (8 variables × 259 respondents). The two component solution explained a total of 66.9% of the variance (Table 2). Rotation was then used to improve the interpretability of the results. Since our variables were correlated, measuring underlying aspects of the same phenomena, we used an oblique rotation [9]. The rotated solution revealed the presence of simple structure, with both components, showing a number of strong loadings and variables loading substantially on one component (Table 3). In Table 3 the loadings of 8 items on the two components are presented. Cut off is set at 0.54.

**Table 3:** Factor loadings of 8 items*. Principal Component Analysis, direct oblique rotation.

Items	Components	

	**1**	**2**

Q1 Municipal health care services become more familiar with how the hospital works.	**.841**	
Q2 Hospital staff learns more from the employees in the municipal health care services.	**.798**	
Q3 Hospital employees have gained a greater understanding of the opinions municipality makes of each patient’s total situation.	**.790**	
Q4 Employees in the municipal health care services learn more from employees of the hospital.	**.772**	
Q5 Municipalities got equal impact for their own interests that the hospital.		**.860**
Q6 A contribution to a more equal relationship between the municipality and the hospital.	.512	**.845**
Q7 The municipality achieved its own local adjustments in the service agreements.		**.773**
Q8 A contribution that municipalities and hospitals jointly ensure overall and continuous health care services.	.534	**.742**

* Items were based on 5-point Likert scales and formulated as statements. (5 = to a great extent, 1 = very little extent). The initial question to item Q1–Q4 was: *To what extent do you perceive that The Norwegian Coordination Reform has led to*. The initial question to item Q5 and Q7 was: *To what extent applied the following in the process when mandatory service agreements were negotiated?* The initial question to item Q6–Q8 was: *To what extent are mandatory service agreements in The Norwegian Coordination Reform*.

There was a weak positive correlation [9] between the two factors (r = .34).

### Step 2

Interpretation of PCA (Table 3) revealed two major patterns (Table 4), where items that constitute component 1 are the most important. We labelled this dimension learning and expertise equality (LEE). The dimension LEE reveals the relative importance of mutual understanding, learning and knowledge sharing, seen from the perspective of municipalities. Dimension two (Table 4) was labelled contractual equality (CE). Prior to The Norwegian Coordination Reform municipalities perceived asymmetry in the formal relation with their partners (hospitals) [8]. Contractual equality reveals the relative importance of mandatory service agreements and perceived fairness vis-à-vis the process which led to these agreements.

**Table 4:** Reliability for two dimensions.

Dimensions of partnership equality		No. of items	Cronbach’s alpha	n =

*Learning and expertise equality (LEE)*		4	.817	211
Q1	Municipal health care services become more familiar with how the hospital works.			
Q2	Hospital staff learns more from the employees in the municipal health care services.			
Q3	Hospital employees have gained a greater understanding of the opinions municipality makes of each patient’s total situation.			
Q4	Employees in the municipal health care services learn more from employees of the hospital.			
*Contractual equality (CE)*		4	.803	142
Q5	Municipalities got equal impact for their own interests that the hospital.			
Q6	A contribution to a more equal relationship between the municipality and the hospital.			
Q7	The municipality achieved its own local adjustments in the service agreements.			
Q8	A contribution that municipalities and hospitals jointly ensure overall and continuous health care services.			

### Step 3

Based on results from step two we composed the additive indexes LEE (learning and expertise equality) and CE (contractual equality). Each index ranges from 4 to maximum 20.

## Results

Results in Table 5 and 6 are above average (≥ 8) both on the LEE- and CE-index, indicating perceived mutuality from the perspective of Norwegian municipalities. Still, municipalities respond that employees in the municipal health care learn more from employees of the hospital, then opposite (Table 5). Results presented in Table 6 indicate that municipalities have experienced relative fairness in relation to hospitals during to prior negotiation processes. We also find that municipalities in general support the reform instrument (mandatory service agreements, Table 6).

**Table 5:** Summary Statistics, Learning and expertise equality (LEE), Mean, Standard Deviations and, Correlations, n = 211.

	Variable*	M	SD	1	2	3	4

1	Hospital staff learns more from the employees in the municipal health care services.	2.24	1.06	1.00	.455	.579	.569
2	Employees in the municipal health care services learn more from employees of the hospital.	2.84	.959	.455	1.00	.551	.451
3	Municipal health care services become more familiar with how the hospital works.	3.00	1.01	.579	.551	1.00	.562
4	Hospital employees have gained a greater understanding of the opinions municipality makes of each patient’s total situation.	2.68	1.02	.569	.451	.562	1.00
LEE additive index		10.77	3.27				

* The initial question to variable 1–4 was: *To what extent do you perceive that The Norwegian Coordination Reform has led to*.

**Table 6:** Summary Statistics, Contractual equality (CE), Mean, Standard Deviations and Correlations, n = 142.

	Variable	M	SD	1	2	3	4

1	The municipality achieved its own local adjustments in the service agreements.	2.46	1.25	1.00	.509	.408	.395
2	The municipalities got equal impact for their own interests as the hospital.	2.75	1.20	.509	1.00	.590	.437
3	A contribution to a more equal relationship between the municipality and hospital.	2.88	1.20	.408	.590	1.00	.717
4	A contribution that municipalities and hospitals jointly ensure overall and continuous health services.	3.05	1.05	.395	.437	.717	1.00
CE additive index		11.15	3.74				

* The initial question to variable 1 and 2 was: To what extent applied the following in the process when mandatory service agreements were negotiated? The initial question to variable 3–4 was: To what extent are mandatory service agreements in The Norwegian Coordination Reform.

## Discussion

A lack of equality on the part of the municipalities was an obstacle for patient centered collaboration in municipal-hospital partnerships prior to the Norwegian Coordination Reform [8,32]. We find similar results in the integrated care literature [4,6,17,21,23]. This suggests a need for research on equality in partnerships within primary care. However, the perspective from hospitals, professionals and patients, would have provided a richer understanding of the investigated phenomena. According to Valentijn et. al. [10] all these perspectives together, contribute to the conceptualisation of integrated care from a primary care perspective.

First, the study shows that the two dimensions “learning and expertise equality” and “contractual equality” collects reliable and valid data to measure some aspects of equality in mandatory partnerships between hospitals and municipalities (Table 4, 5 and 6). The LEE –dimension consists of variables that reflect the findings in previous research, which show that equality between professionals and mutual respect is basic organizational determinants of collaborative practice [36]. Our findings suggest that this is also the case for inter-organizational collaboration. At the same time, the variables constituting the LEE dimension reflect aspects of what Benson [28] refers to as autonomy as a central resource at stake in inter-organizational collaboration. Benson [28] argues that organizations in partnerships tend to defend their domain, knowledge, their way of doing things, and their definition of both problems and solutions. The hospitals’ defence and expansion of their autonomy has been problematic for the municipalities. Our findings reflected in the LEE- dimension, suggest that the municipalities experience that their autonomy is raised due to the implementation of mandatory partnerships. We find similar results in the Danish Structural Reform, where the power relationship between the regional and municipal authorities is described as more equal, after the introduction of mandatory health agreements in 2007 [23].

Secondly, equality is also reflected in the contractual equality dimension. The variables constituting the contractual equality dimension treat equality dimensions of partnerships related to the formalization of the partnership through contract negotiation and signing. Similar to LEE, CE might be interpreted as an improvement in the autonomy of the municipalities. One possible explanation for this, is that the Norwegian municipalities participated in inter-municipal alliances and networks to strengthen their negotiating position [25]. Similar negotiation strategies were used successfully by Danish municipalities in the Structural Reform [23].

Finally, we provide some remarks on voluntarism in mandatory health care partnerships. According to the literature, mutuality and equal relationships between organizations are important for enhancing collaboration [19,34,35]. An ideal model of partnerships and collaboration usually denotes voluntarism as a prerequisite for achieving results [19,20]. Our results indicate that within mandatory partnerships such as the Norwegian example used here, it can be appropriate to equalize partnerships between providers at different care levels to achieve better vertical collaboration in the partnership. In such an equalization process, the formal regulation of the partnership and the way parties mobilize in negotiation is important [27]. In the future, other research designs or methods should be used to study to what degree collaboration outcomes are affected by equality aspects in partnerships. Based on literature, partnerships or networks in integrated care at the macro level seek to affect the structural determinants of health [10,17]. At the organizational level, however, partnerships are aimed at delivering a complex range of health services that are changing, as municipal care and hospitals need change. Hence, there is no guarantee that partnership models will evolve successfully over time.
